# A Late Cretaceous mammal from Brazil and the first radioisotopic age for the Bauru Group

**DOI:** 10.1098/rsos.180482

**Published:** 2018-05-30

**Authors:** Mariela C. Castro, Francisco J. Goin, Edgardo Ortiz-Jaureguizar, E. Carolina Vieytes, Kaori Tsukui, Jahandar Ramezani, Alessandro Batezelli, Júlio C. A. Marsola, Max C. Langer

**Affiliations:** 1 Laboratório de Paleontologia, FFCLRP, Universidade de São Paulo, Ribeirão Preto-SP 14040-901, Brazil; 2 División Paleontología Vertebrados, Facultad de Ciencias Naturales y Museo, Universidad Nacional de La Plata, Paseo del Bosque S/N°, B1900FWA La Plata, Argentina; 3 LASBE, Facultad de Ciencias Naturales y Museo, Universidad Nacional de La Plata, Paseo del Bosque S/N°, B1900FWA La Plata, Argentina; 4 División Zoología Vertebrados, Facultad de Ciencias Naturales y Museo, Universidad Nacional de La Plata, Paseo del Bosque S/N°, B1900FWA La Plata, Argentina; 5 CONICET, Buenos Aires, Argentina; 6 Department of Earth, Atmospheric and Planetary Sciences, Massachusetts Institute of Technology, Cambridge, MA 02139, USA; 7 Department of Geology and Natural Resources, IG, Universidade Estadual de Campinas, Campinas-SP, Brazil

**Keywords:** Tribosphenida, enamel reduction, Bauru Basin, South America, U-Pb geochronology, Mesozoic

## Abstract

In the last three decades, records of tribosphenidan mammals from India, continental Africa, Madagascar and South America have challenged the notion of a strictly Laurasian distribution of the group during the Cretaceous. Here, we describe a lower premolar from the Late Cretaceous Adamantina Formation, São Paulo State, Brazil. It differs from all known fossil mammals, except for a putative eutherian from the same geologic unity and *Deccanolestes hislopi*, from the Maastrichtian of India. The incompleteness of the material precludes narrowing down its taxonomic attribution further than Tribosphenida, but it is larger than most coeval mammals and shows a thin layer of parallel crystallite enamel. The new taxon helps filling two major gaps in the fossil record: the paucity of Mesozoic mammals in more northern parts of South America and of tribosphenidans in the Cretaceous of that continent. In addition, high-precision U-Pb geochronology provided a post-Turonian maximal age (≤87.8 Ma) for the type stratum, which is overlain by the dinosaur-bearing Marília Formation, constraining the age of the Adamantina Formation at the site to late Coniacian–late Maastrichtian. This represents the first radioisotopic age for the Bauru Group, a key stratigraphic unit for the study of Cretaceous tetrapods in Gondwana.

## 1. Introduction

The mammalian clade Tribosphenida [1] (=Boreosphenida [2]) includes eutherians and metatherians, with records as old as the Early Cretaceous (Berriasian) in the Northern Hemisphere [3]. The group is characterized by the presence of a prominent protocone occluding on a basined talonid, combining shearing and grinding in a single chewing stroke [4,5]. In the last three decades, records from India [6–8], Madagascar [9,10], continental Africa [11] and South America [12–14] have challenged the notion that the group was restricted to Laurasia during the Cretaceous. Unfortunately, despite few exceptions like the Indian *Deccanolestes hislopi* [15,16], these occurrences are fragmentary and/or scarce, hampering more precise phylogenetic placements.

**Figure 1.**
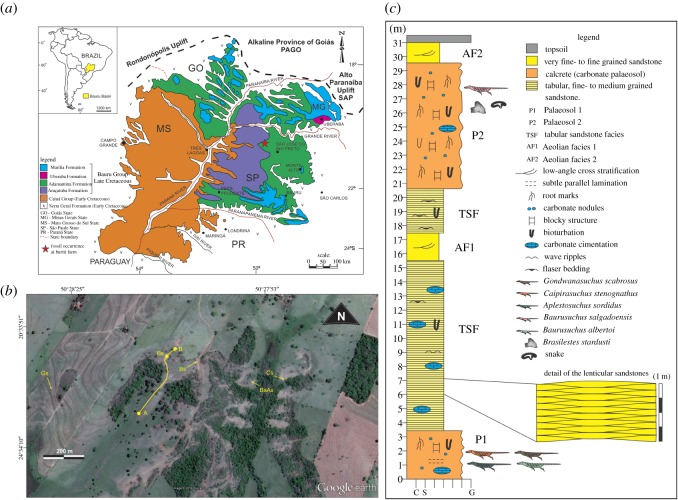
Geological settings. (*a*) Surface distribution map of Bauru Basin stratigraphic units (modified from ref. [17] using CorelDRAW 2017 www.coreldraw.com); (*b*) Google Earth image (2016) marking fossil occurrences at Buriti Farm, São Paulo State, Brazil; (*c*) Stratigraphic column of the studied site. A–B, transect of the stratigraphic column; BaAs, *Baurusuchus albertoi* and *Aplestosuchus sordidus* type-locality; Bs (within the A–B transect), *B. stardusti* type-locality; Bs, *Baurusuchus salgadoensis* type-locality; Cs, *Caipirasuchus stenognathus* type-locality; Gs, *Gondwanasuchus scabrosus* type-locality.

Here, we describe a new mammal species based on an isolated tooth collected during 2015 (in accordance with Brazilian laws) from the Late Cretaceous Adamantina Formation, in the area of General Salgado, São Paulo State, Brazil (figure 1; electronic supplementary material, figures S4–S7). We also provide the first radioisotopic age for Bauru Group deposits, which is an important window to the biodiversity of Western Gondwana in the Late Cretaceous, especially due to its tetrapod records.

## 2. Material and methods

### 2.1. U-Pb geochronology

Zircons were isolated from an approximately 1 kg sample of the bed where the mammalian remain was collected using standard crushing, as well as magnetic and density separation techniques. Preliminary analyses of selected zircons (z1 to z17) from this sample by the high-precision chemical-abrasion isotope-dilution thermal-ionization mass spectrometry (CA-ID-TIMS) technique at MIT revealed a wide range of detrital zircon ages from Neoproterozoic to *ca* 87.8 Ma. To isolate the youngest population of zircons that would most closely approximate the depositional age, 100 additional zircon grains were placed in a grain mount and analysed by laser-ablation inductively coupled plasma mass spectrometry (LA-ICP-MS) at the University of Kansas, following the procedures described in Cioffi *et al*. [18]. Based on the LA-ICP-MS results, seven youngest dated zircons were removed from the mount and subsequently analysed by the CA-ID-TIMS method at MIT.

The CA-ID-TIMS analyses were carried out at the MIT Isotope Lab following the analytical procedures described in Ramezani *et al*. [19]. The selected grains were pre-treated by a chemical-abrasion method modified after Mattinson [20] involving thermal annealing at 900°C for 60 h and leaching in concentrated HF at 210°C for 12 h in order to mitigate the effects of radiation-induced Pb loss in zircon. The EARTHTIME mixed ^205^Pb–^233^U–^235^U (ET535) tracer [21,22] was used in the analyses and isotopic measurements were made either on the VG Sector 54 or on Isotopx X62 multi-collector mass spectrometers equipped with Daly photomultiplier ion-counting systems at MIT. Reduction of mass spectrometric data, as well as calculation of dates and propagation of uncertainties, was done using the Tripoli and U-Pb_Redux software and associated algorithms [23,24].

Complete U-Pb data are given in electronic supplementary material, tables S1 (ICP-MS) and S2 (ID-TIMS), and the analyses used in age calculation are illustrated in the date distribution plot of electronic supplementary material, figure S8. All tabulated U-Pb data are reported with 2*σ* analytical (internal) uncertainties, whereas the maximum depositional age stated in the text includes all sources of uncertainty. The youngest measured ID-TIMS ^206^Pb/^238^U dates are considered a maximum constraint on the age of deposition of the corresponding bed and its associated *in situ* fauna.

### 2.2. Enamel analyses

The labial face of the tooth and the tip of the main cusp were prepared for enamel study according to the protocol described in Flynn & Wahlert [25]. Quantitative microanalyses on the uncoated premolar were performed by energy-dispersive X-ray spectrometry (EDS), using a JEOL 6510 LV scanning electron microscope (SEM) coupled to Oxford Instrument X-Max EDS detector, operating at 30 kV.

## 3. Results

### 3.1. U-Pb geochronology

The LA-ICP-MS ^206^Pb/^238^U dates have an average 2*σ* uncertainty of 2.6% and range from approximately 3.3 Ga to 85.2 ± 2.7 Ma, indicating a highly mixed detrital zircon population (see electronic supplementary material). The ID-TIMS ^206^Pb/^238^U dates are characterized by 2*σ* uncertainties of the order of 0.2% and range from 620.4 ± 1.6 Ma to 87.782 ± 0.062 Ma. The youngest two high-precision ID-TIMS analyses (z17 and z18*) overlap within uncertainty (and with the youngest five LA-ICP-MS dates), probably representing the youngest zircons that exist in the sample (electronic supplementary material, figure S8). The data are not sufficient to calculate a weighted mean date; therefore, the youngest ID-TIMS date of 87.782 ± 0.062 Ma (±0.12 Ma including total propagated uncertainties) best represents the maximum age of deposition of the fossil-bearing bed.

### 3.2. Systematic palaeontology

Tribosphenida McKenna 1975

**Figure 2.**
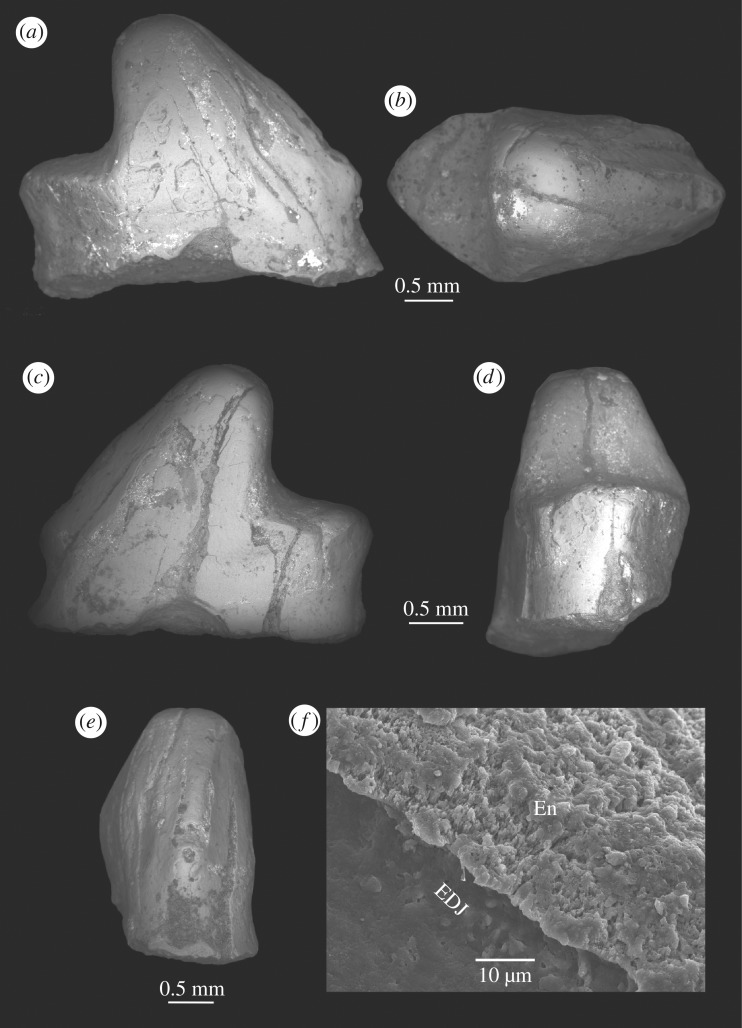
*Brasilestes stardusti* gen. et sp. nov. (LPRP/USP 0751, holotype). Lower right premolar (probably p3 or p4) in different views: (*a*) labial; (*b*) occlusal; (*c*) lingual; (*d*) posterior; and (*e*) anterior; (*f*) thin enamel layer, with no prisms detected, on the labial side of the tooth. En, enamel; EDJ, enamel–dentine junction.

*Brasilestes stardusti* gen. et sp. nov.

(figure 2; electronic supplementary material, figures S1–S3)

**Etymology**. The genus name combines the words Brasil and *ληστ*ής (Greek for robber). Species epithet after the fictional character Ziggy Stardust, honouring the deceased artist David Bowie.

**Holotype.** LPRP/USP 0751, isolated lower right premolar, probably p3 or p4 (figure 2).

**Type locality and horizon.** Buriti Farm, General Salgado, São Paulo, Brazil (20°33'56′′ S, 50°28'09′′ W); Adamantina Formation, Bauru Group, Bauru Basin (figure 1).

**Age.** High-precision U-Pb geochronology of detrital zircons from the fossil-bearing bed yielded a maximum depositional age of 87.78 ± 0.12 Ma (see electronic supplementary material).

**Diagnosis.** *Brasilestes stardusti* differs from other described mammals by a unique suite of traits of its two-rooted premolar: strongly asymmetric profile outline; single and slightly recumbent main cusp, with an anterior crest and a flat posterior surface; small anterior cuspule; flat talonid, perpendicular to the posterior surface of the main cusp, corresponding to one-third of the total length of the crown; extremely thin enamel layer (less than 20 µm).

**Description.** LPRP/USP 0751 is large (max. crown length = 3.5 mm; max. crown width = 1.8 mm) compared to most Mesozoic mammal premolars. Although broken at the root–crown junction, the tooth is clearly two-rooted and its outline is strongly asymmetric in the lateral view. It has a single, slightly posteriorly recumbent main cusp, with an anterior crest and a flat posterior surface perpendicular to a relatively wide talonid. Although the apex is worn, the anterior and posterior borders of the main cusp suggest it was pointed. The anterior crest ends at the base of the crown in a barely differentiated, very small cuspule that defines the anterior-most end of the crown. A somewhat concave vertical facet is seen in the mesio-lingual surface of the main cusp. No posterior cingulum is present. The labial surface is more salient and more convex at the contact between the main cusp and the talonid, whereas the lingual surface is flatter. The talonid corresponds to approximately one-third of the total length of the crown. It has a nearly triangular outline in the occlusal view, narrowing towards the posterior end, and bears no cusps. Its surface is flat, with signs of wear. A notch marks a tiny cuspule at the lingual border, halfway between the posterior end of the tooth and the posterior surface of the main cusp. The preserved morphology suggests there were well-developed roots, the anterior slightly larger than the posterior.

**Enamel microstructure and EDS analyses** (figure 1, figures S1–S3): the enamel layer is extremely thin (less than 20 µm thick); it does not homogeneously cover the surface of the crown, being absent in some areas (mostly at the tip and the anterior slope of the main cusp). As the enamel shows numerous cracks, we regard these absences as due to post-mortem processes (probably spalling of the tooth exposed to sub-aerial weathering and/or desiccation; [26]). Processes of enamel loss or thinning due to digestion by predators and abrasion during transport were discarded (see below). Regarding its microstructure, the enamel is prismless, being composed of crystals that are displayed parallel to each other and lie perpendicular to the enamel–dentine junction (EDJ). The columns that characterize the Synapsid Columnar Enamel were not observed.

The results of the elemental analyses (electronic supplementary material, figure S3) are similar to those of previous studies on fossil bones and teeth from several fossil sites [27,28]. The major elements include oxygen (35.6 wt%), calcium (29.6 wt%) and phosphorus (10.2 wt%). These are components of hydroxyapatite, the main mineral that forms dental enamel and dentine, and may correspond to the original composition of the tooth. The calcium could also have been originated from paedogenetic precipitation of calcium carbonate during the calcrete formation (see above). Likewise, this paedogenic event could be responsible for the great amount of carbon in the sample (19.9 wt%). Among the minor elements, magnesium (0.5 wt%), sodium (0.4 wt%), potassium (0.2 wt%) and chlorine (0.1 wt%) may also compose the dental tissues [29]. Silicon (1.1 wt%) and iron (0.5 wt%), as well as the aluminium (0.97 wt%) in lesser degree, are more concentrated in the striations on the surface, where the sediment is accumulated, and might be related to oxidation processes during the current paedogenesis.

## 4. Discussion

### 4.1. Age of the fossil bed

The Adamantina and correlated formations can be conservatively considered younger than the underlying Valanginian-Barremian Serra Geral basalts (part of the Paraná-Etendeka Volcanic Province, ^40^Ar-^39^Ar dated at *ca* 137–127 Ma; [30]) and older than the Cenozoic, as they are covered over nearly the entire Bauru Basin by the dinosaur-bearing beds of the Marília Formation (usually accepted as Maastrichtian in age, based on both vertebrates and microfossils; [31–33]). As such, the maximum depositional age of 87.78 ± 0.12 Ma provided by the U-Pb zircon geochronology (see above) further constrains the age of the Buriti Farm fossil site as late Coniacian to late (although probably not the latest) Maastrichtian. Previous age estimates for the Adamantina Formation were biochronology-based and rather inconsistent. Based on ostracods and charophytes, a Turonian–Santonian age has been suggested [31,33], but a contrasting Campanian–Maastrichtian age, also based on ostracods and charophytes, was proposed by Gobbo-Rodrigues *et al*. [34] and corroborated by the record of sauropod dinosaurs ([32], but see [33]). Finally, a broader Cenomanian–Campanian age range was proposed based on a review of the collective fossil content of the Adamantina and correlated formations [35].

The correlation data provided by fossils of the Buriti Farm are inconclusive. The dinosaur and snake remains are either too fragmentary or poorly studied to allow a precise taxonomic identification. Sphagesaurids are largely endemic to the Bauru Basin and the only exception, *Yacarerani boliviensis* [36], comes from the presumably Late Cretaceous (Maastrichtian) Cajones Formation of Bolivia [37]. Likewise, well-known Baurusuchinae, the clade that includes three of the four baurusuchids of the Buriti Farm, are restricted to the Adamantina Formation [38]. As for *Gondwanasuchus scabrosus*, it derives from an earlier phylogenetic split within Baurusuchidae [38], and could potentially indicate an older age.

Our new chronostratigraphy is in agreement to some of the previous age estimates [31,33,34], but rules out any pre-Coniacian age for the *B. stardusti* fossil bed. In addition, magnetostratigraphic studies in the Uberaba Formation [39], an inferred lateral correlate of the Adamantina Formation [17], indicate that it is younger than the Cretaceous Normal Superchron (*ca* 121–83 Ma; [40]). Such a Campanian maximal age fits the results of the U-Pb geochronology presented here, and could be tentatively extended to the Adamantina Formation, following its inferred synchronicity to the Uberaba Formation. Yet, it is important to consider that previous workers assigned ages to the Adamantina Formation and its set of possible correlates, like the Uberaba Formation, as a whole, but there is presently no evidence that these stratigraphic units could not actually span a significant portion of their assigned age bracket. Further stratigraphic and geochronologic work is needed to fully understand the depositional history of the Bauru Basin and its extensive fossil record.

### 4.2. Enamel

The presence of a thin layer of enamel partially covering the premolar of *B. stardusti* is particularly interesting. As mentioned, the absence of enamel on the main cusp, as well as the numerous cracks on the dental surface, could be a result of spalling related to sub-aerial weathering and desiccation [26]. On the other hand, the specimen does not show signs of digestion by predators as a cause of the loss or thinning of the enamel. Considering the fossil fauna of the locality (see above), the most likely predator of a small- to medium-sized mammal like *Brasilestes* would be a crocodile. The crocodile remains are abundant in the Bauru Group and the described forms (approx. 25 species; [41]) range from 0.5 to 4 m long, approximately. The digestion in modern crocodiles decalcifies tooth tissues, completely removing the enamel (except by residual interdental patches), leaving the organic matrix of the dentine and cement, which decompose within days under aerobic conditions [26]. The presence of a tooth with corroded enamel in a crocodylomorph coprolite from the Bauru Group illustrates such a process [42]; this is not the case of specimen LPRP/USP 0751. Other possible predators in the site would be medium-sized avian and non-avian theropods, but their remains are relatively scarce in the Bauru Group [43,44]. Digestion by birds (except falconiforms) and mammals does not significantly alter the enamel distribution [26]. Yet, artificial digestion of teeth under experimental conditions shows that digestion corrodes the enamel in a characteristic irregular pattern [45], different from the one observed in *Brasilestes*.

### 4.3. Affinities of *Brasilestes stardusti*

*Brasilestes stardusti* was compared to a wide range of Mesozoic and Cenozoic species (see electronic supplementary material, for an expanded comparison). It differs from nearly all of them, except for two eutherians: a possible placental briefly described from the same geological unit as *B. stardusti* [13] and *Deccanolestes hislopi* from the Maastrichtian of India [6], as detailed below.

The main differences of the posterior lower premolars in the analysed taxa relative to that of *B. stardusti* are an incipient to absent basined talonid and/or the presence of more cusps. This is the case of Australosphenida (see below), Eutriconodonta, Allotheria, ‘Symmetrodonta’ and ‘Eupantotheria’ (including Dryolestoidea, Meridiolestida and Zatheria). Some deciduous premolars of *Nanolestes* have a posterior talonid as developed as in *B. stardusti*, but are anteriorly multicuspate and have a basin among the cusps [46]. In the early tribosphenidan *Slaughteria,* p3 has a talonid on the posterior third of the tooth, but it ends in a posterior cusp and a labial cuspule, whereas the main cusp is flanked by an anterior and a posterior crest; dp4 and dp5 are molariforms with fully developed trigonids [5]. The posterior premolars of metatherians differ from that of *B. stardusti* because they are labiolingually compressed and relatively more symmetric (Didelphimorphia, Paucituberculata, Microbiotheria, Sparassodonta and Dasyuromorpha), or they are robust, molariform or have plagiaulacoid aspect (Polydolopimorphia and Diprotodontia). The Australian marsupial *Naboryctes* shows asymmetric posterior premolars, but the main cusp is procumbent and the incipient talonid ends in a cusp. Among Eutheria, most taxa have either labiolingually compressed teeth, no or incipient talonid, more cusps/crests than *B. stardusti*, or (sub)molariform lower premolars. In *Gypsonictops*, although p4 has a talonid and is asymmetric in lateral view as in *B. stardusti*, there is an anterior cingulum, one to three posterior cuspules, and the posterior wall of the main cusp develops into a crest [47].

As for the reduced enamel, when present in Palaeanodonta this tissue is composed of a relatively thin layer (70–140 µm; [48]). However, the enamel is much thicker than that of *B. stardusti*, as well as prismatic, and the morphology of their posterior lower premolars differs in having either more crests/cusps, or absent/incipient talonid (e.g. *Ernanodon* [49,50], *Melaniella* [51], *Tubulodon* [52]). In *Mylanodon*, p4 develops a talonid that narrows posteriorly, as in *B. stardusti*, but it lacks an anterior crest and the main cusp shows a vertical lingual groove [53].

In Xenarthra, even though most taxa completely lack enamel, a thin layer is retained in the extinct armadillos *Astegotherium* and *Utaetus*, and in the extant long-nosed armadillo *Dasypus* [54], but their homodont dentition is composed of subcylindric teeth. Work in progress on the microstructure of permanent molars of *Dasypus* (M. Ciancio, E.C.V., M.C.C. and A.A. Carlini, in preparation) shows a thin enamel layer (less than 25 µm) with or without prisms depending on the species, a condition strikingly similar to that of *B. stardusti* (see electronic supplementary material, figure S2). Different lines of evidence indicate that xenarthrans had a South American origin no younger than 85 Ma [55,56], but the oldest fossils of the group come from the early Palaeogene [56]. Although the age and provenance of *B. stardusti* (Coniacian–Maastrichtian of tropical South America) match previous molecular hypotheses for the origin of Xenarthra, inference of taxonomic affinity is premature in the face of the morphologic differences, scarcity of remains and plasticity of enamel reduction among different mammalian lineages.

**Figure 3.**
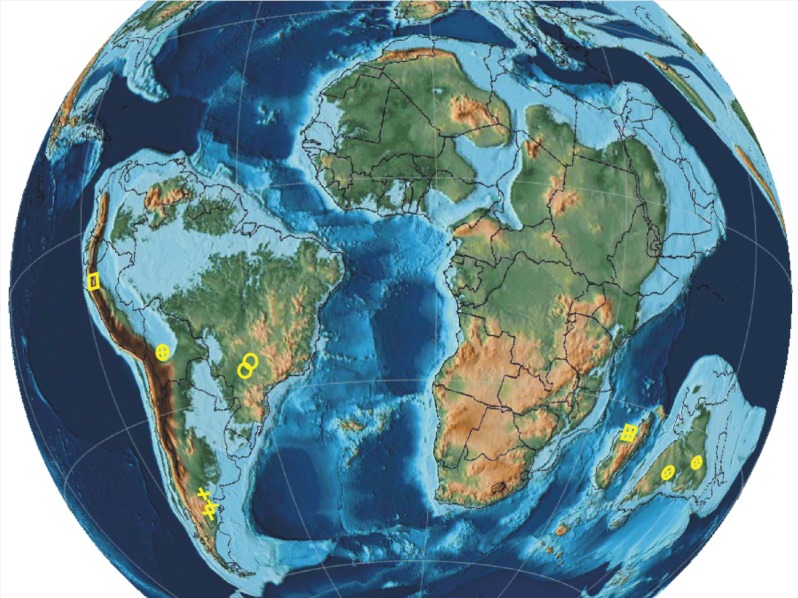
Distribution of Gondwanan landmasses in the Late Cretaceous (80 Ma) and known mammalian records. Circle, eutherian; square, indeterminate tribosphenidan; plus, non-tribosphenidan (map generated using the softwares GPlates 2.0.0 www.gplates.org and PaleoData Plotter https://www.earthbyte.org/paleomap-paleoatlas-for-gplates; [66]).

Conspicuous similarities were found only between *B. stardusti* and two other taxa, both referred to Eutheria. The first is *Deccanolestes hislopi* (Maastrichtian of India), particularly the p3 figured in the original description [6] (fig. 3, NKIM 12; also in [57], Plate III). As in *B. stardusti*, there is a clear asymmetry between the anterior and the posterior halves, a single large cusp, and a small notch in the lingual border of the talonid delimiting a minute cuspule. In fact, *B. stardusti* is more similar to that specimen of *D. hislopi* (NKIM 12) than to any other material of our knowledge. They differ on the talonid, which is flatter in *B. stardusti*, on the angle formed by the posterior surface of the main cusp and the talonid (perpendicular in *B. stardusti* and obtuse in *D. hislopi*), and on the presence of one or two talonid cusps in the p2 and p3 of *D. hislopi* [58]. Also, there is a considerable difference in the size of both teeth: the p3 of *D. hislopi* is 0.41 mm long, whereas the premolar of *B. stardusti* is 3.5 mm long. Among other species of the genus, *D. robustus* has larger teeth (the m1 is approximately 1.5 mm long; [59]), but still much smaller than that of *B. stardusti*. *Deccanolestes* has been regarded as an Adapisoriculdae, a group that is considered either a non-placental Eutheria or an Euarchonta [15,16,60,61].

Similarities are also found between *B. stardusti* and a possible Placentalia that was previously collected in the Adamantina Formation [13], especially in terms of the talonid development. However, besides the size difference (the length of that p3 is 1.2 mm), its talonid ends in a posterior cusp. Also, the main cusp has an anterior and a posterior crest, is proportionally smaller, and more labiolingually compressed than that of *B. stardusti*.

The incompleteness of *B. stardusti* precludes a more precise taxonomic assignment. As for the inclusion of the taxon in a cladistic framework, the data matrix of [62], for example, focuses on South American Mesozoic mammals and presents 317 craniodental characters. Out of those, the characters that can be scored for *B. stardusti* (i.e. related to isolated premolars) refer to the morphology of the penultimate (characters 43 and 149) or the ultimate (characters 44, 45, 47, 48, 50–52 and 150) lower premolar. Although we infer that *B. stardusti* is represented by a posterior lower premolar, the total number of premolars and the exact locus occupied by this tooth are uncertain; thus, the inclusion of *B. stardusti* in the available phylogenetic studies will be of limited use regarding the inference of its taxonomic affinities.

The diagnoses of Tribosphenida (=Boreosphenida; see [4,5]) and Australosphenida presented by Luo *et al*. [2] include features of the last lower premolar. This tooth is defined as labiolingually wide in the posterior part and having a fully triangulated trigonid in Australosphenida, whereas those features are absent in Tribosphenida. Also, an asymmetric main cusp is not observed in the last premolar of australosphenidans [2], a condition present in *B. stardusti*. In this scenario, as determining the position of its premolar in the lower tooth row is unfeasible, the affinity of *B. stardusti* with Australosphenida cannot be completely ruled out. However, based on the morphological features described above and on the similarities with two purported eutherians, we attribute *B. stardusti* to Tribosphenida.

### 4.4. Biogeographic implications

The evolutionary history of mammals during the Late Cretaceous–Early Palaeocene in South America [63] is marked by the almost complete extinction of endemic non-tribosphenidan lineages (leading to the end of the ‘Gondwanan Episode’) and the expansion of Tribosphenida (the so-called South American Episode; [64]). This remarkable change in land-mammal communities (First Great Turnover) is, however, based primarily on the well-studied Late Cretaceous faunas of Patagonia [64]. Along with previous records of putative tribosphenidans in Peru [12], Bolivia [14] and Brazil [13], *B. stardusti* partially fills two major gaps: the paucity of mammals in more northern parts of South America and of tribosphenidans in the Cretaceous of the continent [65].

During the Late Cretaceous, South America was divided into northeastern and southwestern portions by a sea corridor ([63,65,66]; figure 3), which probably acted as a barrier or filter for some organisms. Moreover, throughout this period, those regions had different climatic conditions, with the warm-temperate Patagonia separated from the arid areas of central and northeastern South America by a subtropical seasonal dry belt [67,68]. Indeed, the Late Cretaceous–earliest Palaeocene terrestrial biotas of Patagonia have greater similarity to those of East Gondwana (i.e. Antarctica and Australia), whereas the faunal assemblage of northern South America suggests a closer biogeographic relation with Africa [63,65].

*Brasilestes stardusti* and the three other records mentioned above [12–14] indicate the presence of tribosphenidans in the Late Cretaceous of northern South America (figure 3). Biogeographically, these tribosphenidans may have either dispersed from the North American part of Laurasia during the Late Cretaceous (as part of the so-called First American Biotic Interchange; [65]), or they may have had a Gondwanic origin [69].

The presence of northern immigrants like hadrosaurs and probably marsupials in South America [65] and, conversely, of southern immigrants like titanosaurs in North America [70], has been explained by a transient inter-American connection during the Late Cretaceous. In this biogeographic context, *B. stardusti* and the other tribosphenidans recorded in northern South America (figure 3) could be interpreted as mammals with a northern ancestry. The tribosphenid mammals of Peru and Bolivia have been considered as related to North American immigrants (see [3] and references therein), but *B. stardusti* raises more questions. If *B. stardusti* is related to Adapisoriculidae (as inferred for *Deccanolestes*) or to Xenarthra (hinted by the reduced enamel), the lack of comparable coeval Laurasian forms is indirect evidence against the hypothesis that the taxon had a northern ancestry. According to the known fossil record, the oldest adapisoriculids have been recorded in Late Cretaceous beds of India [60] and different lines of evidence indicate that xenarthrans had a South American origin no younger than 85 Ma [55,71,72].

Consequently, the hypothesis of a Gondwanan ancestry of *B. stardusti* appears to be better supported than the Laurasian one. Another indirect evidence of a potential Gondwanan origin of *B. stardusti* comes from the record of the advanced theriiform *Vincelestes neuquenianus* in the Early Cretaceous of Patagonia. In spite of *V. neuquenianus* not being closely related to therians (e.g. [73]), its record opens the possibility that forms like *B. stardusti* in northern South America, the adapisoriculid *Deccanolestes* in India or, perhaps, xenarthrans in South America, could represent late and derived survivors of that theriiform lineage in Gondwana.

A more precise taxonomic placement of *B. stardusti* depends upon additional discoveries in the Bauru Basin, but this finding underscores the importance of northern South America in further understanding the history of mammals in Gondwanan landmasses during the Late Cretaceous.

## 5. Conclusion

Based on an isolated lower premolar, *B. stardusti* represents a new tribosphenidan mammal, a rare group in Gondwanan deposits of Cretaceous age. The tooth is larger than those of most coeval mammals, but the definition of more precise taxonomic affinities is hampered by the incompleteness of the type-specimen. It shows a thin layer of parallel crystallite enamel, as previously unrecognized among Cretaceous tribosphenidans. Geologic interpretation of the fossil site indicates intermittent sedimentation events, probably in fluvial environments under a semi-arid climate, and low transport of bioclasts. The maximum age of its type-stratum is constrained by a high-precision U-Pb geochronology as late Coniacian age and by the superposed dinosaur-bearing beds of the Marília Formation, indicating a late Coniacian to late (although probably not latest) Maastrichtian time range for the Adamantina Formation. The occurrence of *B. stardusti*, along with two morphologically similar forms, a putative eutherian from the Adamantina Formation and *Deccanolestes hislopi*, from India, indicates the existence of a still poorly sampled tribosphenidan diversity in the Late Cretaceous of Gondwanan landmasses.

## Supplementary Material

Castro et al_ESM-v3.docx

## Data accessibility

The datasets supporting this article have been uploaded as part of the electronic supplementary material, including: expanded comparison of *Brasilestes stardusti*, additional data on the enamel, geological settings and U-Pb geochronology.
