# Supplementary material for: A Late Cretaceous mammal from Brazil and the first radioisotopic age for the Bauru Group
Source: R Soc Open Sci. 2018 May 30;5(5):180482. doi: 10.1098/rsos.180482 (PMC5990825; doi:10.1098/rsos.180482)
Supplement: Castro et al_ESM-v3.docx [file rsos180482supp1.docx]

Electronic Supplementary Material for:

A Late Cretaceous mammal from Brazil and the first radioisotopic age for the Bauru Group

Mariela C. Castro^1*^, Francisco J. Goin^2,3^, Edgardo Ortiz-Jaureguizar^3,4^, E. Carolina Vieytes^3,5^, Kaori Tsukui^6^, Jahandar Ramezani^6^, Alessandro Batezelli^7^, Júlio C. A. Marsola^1^, Max C. Langer^1^

*^1^ Laboratório de Paleontologia, FFCLRP, Universidade de São Paulo, Ribeirão Preto-SP, 14040-901, Brazil.*

*^2^ División Paleontología Vertebrados, Facultad de Ciencias Naturales y Museo, Universidad Nacional de La Plata, Paseo del Bosque S/Nº, B1900FWA La Plata, Argentina.*

*^3^ CONICET*

*^4^ LASBE, Facultad de Ciencias Naturales y Museo, Universidad Nacional de La Plata, Paseo del Bosque S/Nº, B1900FWA La Plata, Argentina.*

*^5^ División Zoología Vertebrados, Facultad de Ciencias Naturales y Museo, Universidad Nacional de La Plata, Paseo del Bosque S/Nº, B1900FWA La Plata, Argentina.*

*^6^ Department of Earth, Atmospheric and Planetary Sciences, Massachusetts Institute of Technology, Cambridge, Massachusetts 02139, USA.*

*^7^ Department of Geology and Natural Resources, IG, Universidade Estadual de Campinas, Campinas-SP, Brazil.*

**This file includes:**

1. Expanded comparison of *Brasilestes stardusti*.

2. Additional data on the enamel.

3. Geological settings.

3.1. Type-locality and -section

3.2. Palaeoenvironmental interpretations

4. U-Pb geochronology.

5. Supplementary Information References

**1. Expanded comparison of** ***Brasilestes stardusti***

A non-exhaustive comparison between *Brasilestes* and named mammalian taxa is presented below. Except when specified, the classification of the major groups follows ref. [3]; among eutherians, the systematics follows ref. [74].

Australosphenida and *Shuotherium*: where known, the ultimate lower premolars of Australosphenida have a strongly molarized trigonid, little to no talonid, and mesial cingulid, synapomorphies that may be shared with *Shuotherium* (see [3]). In *Ambondro* (middle Jurassic of Madagascar) the ultimate premolar is present in the holotype and shows a main cusp flanked by small accessory cusps, and a labial cingulum [75]. Similarly, the p5 in the holotype of *Ausktribosphenos* (early Cretaceous of Australia) exhibits three cusps and cingula [76]. *Bishops* (early Cretaceous of Australia) has simpler premolars, labiolingually compressed, and a small cuspule may be present posterior to the main cusp, at the base of the crown [77]. The premolars of *Asfaltomylos* (middle Jurassic of Argentina) are poorly preserved but, based on the p?5 of the holotype, seem to be antero-posteriorly symmetrical [78]. In *Henosferus* (Jurassic of Argentina) the premolar are narrow, more symmetrical, and lack talonid [4]. The cretaceous monotremes have no premolar known, except for the ultimate superior premolar of *Kollikodon* (early Cretaceous of Australia), which has a main cusp and an anterior cuspule, lacking an anterior crest [3]. The p1-p3 of *Shuotherium (*middle–late Jurassic of England and China) are extremely compressed labiolingually, with anterior and posterior crests flanking the pointed main cusp, whereas p4 has a fully triangulated trigonid [79].

Eutriconodonta: present multicuspate premolars with no talonids.

Allotheria (*sensu* ref. [80]): the premolars are multicuspate and proportionally wider. When there is a specialization, the p4 is extremely compressed labiolingually and the p3 is simplified or absent. Although there is not a differentiation between premolars and molars in Gondwanatheria, the more anterior postcanine teeth are completely molariform; also, the enamel layer is thick.

"Symmetrodonta": the premolars are trenchant, usually dominated by a large main cusp and small anterior and posterior cuspules, with a poorly developed or absent talonid.

“Eupantotheria” (Stem Cladotherians): some dryolestoids (e.g., *Dryolestes leiriensis*, from the late Jurassic of Portugal) show premolars that are superficially similar to *Brasilestes*, with a large and slightly recumbent main cusp and an anterior crest of variable development; nonetheless, they are less antero-posteriorly asymmetric and lack talonid. In other cases, the dp3 and dp4 may be molariform, consisting of a trigonid and a small, single-cusped talonid [81]. In *Cronopio* (late Cretaceous of Argentina) the lower premolars are relatively symmetric in labial view and show a posterior cuspule, with no development of a talonid. Premolars of *Peligrotherium* (early Palaeocene of Argentina) are bulbous, with low and blunt cusps [82]. In *Necrolestes* (early Miocene of Argentina), p1 is tricuspid, whereas p2 and p3 are fully molarized [62]. Premolars of other Meridiolestida (*sensu* ref. [83]) are not known. The p1 of *Vincelestes* (early Cretaceous of Argentina) is diminutive, tricuspate, and antero-posteriorly symmetric; the anterior and the posterior portions of p2 are isodiametric, and a posterior crest is present. Among the Zatheria, the preserved lower premolars lack talonid and show an anterior cuspule. Some deciduous premolars of *Nanolestes* (late Jurassic of China and Portugal) present a posterior talononid as developed as in *Brasilestes*, but are anteriorly multicuspate and have a basin among the cusps [84].

“Tribotheria” (Stem Boreosphenidans): as represented by *Slaughteria* (early Cretaceous of the United States; premolars in other boreosphenidans are scarce), the p3 has a talonid on the posterior third of the tooth, but it ends in a posterior cusp and a labial cuspule, whereas the main cusp in flank by an anterior and a posterior crest; the dp4 and dp5 are molariforms with fully developed trigonids [85].

Metatheria: any metatherians show a premolar morphology similar to *Brasilestes*. The premolars of Didelphimorphia, Paucituberculata, Microbiotheria, Sparassodonta, and Dasyuromorpha are labiolingually compressed and relatively more symmetric. In several linages of Polydolopimorphia, the posterior premolars are robust and, in some cases, have plagiaulacoid aspect. Among the Australian marsupials, the early Notoryctemorphia *Naraboryctes* (early Miocene of Australia) clearly shows asymmetric posterior premolars, but the main cusp is procumbent and the incipient talonid ends in a cusp. The lower premolars have molariform or plagiaulacoid aspect among the Diprotodontia.

Eutheria: several stem eutherians present labiolingually compressed lower premolars, without talonid or with an incipient one; also, an anterior and a posterior cusp, of variable developments, usually flank the main cusp. This is the case of *Juramaia* (late Jurassic of China) [73] and *Prokennalestes (*early Cretaceous of Mongolia) [86; 87]. Additionally, the premolars of *Eomaia* (early Cretaceous of China) are more symmetric than seen in *Brasilestes*. In other cases (e.g. *Montanalestes*; , from the early Cretaceous of the United States [88]) the ultimate premolar is molariform.

Among the Zhelestidae, the p4 of *Aspanlestes* presents a main cusp with a strong posterior crest and an incipient talonid ending in a posterior cusp; the p5 is submolariform [89].

The p4 and p5 of *Paranyctoides* (late Cretaceous of Uzbekistan, Canada and the United States) show a well-defined anterior cusp, posterior crest(s) in the main cusp, and a posterior talonid cusp [90; 91].

Among the Cimolestidae, *Cimolestes* (late Cretaceous of Canada and the United States, and Palaeocene of Belgium, Bolivia, Canada, Morocco, and the United States) has strongly labiolingually compressed premolars that end in a well-defined posterior cusp [47]. The same is observed in the p4 and p5 of *Maelestes* (late Cretaceous of Mongolia), which also show a small antero-basal cusp [74].

Among the Asioryctitheria, *Kennalestes* (late Cretaceous of Mongolia and Uzbekistan) has the entire premolar series strongly labiolingually compressed, with an anterior main cusp, an unbasined heel, and a posterior cusp [92].

We found notorious similarities between *Brasilestes* and the p3 referred to *Deccanolestes* (late Cretaceous of India) in the original description (ref. [6], Fig. 3, NKIM 12; also figured in ref. [93], Plate III). In both teeth there is a clear asymmetry between the anterior and the posterior halves, a single large cusp, and a small notch in the lingual border of the talonid delimiting a diminutive cuspule. In fact, *Brasilestes* is more similar to that specimen of *Deccanolestes* (NKIM 12) than to any other material compared by us. They differ on the talonid, which is flatter in *Brasilestes*, and on the angle formed by the posterior surface of the main cusp and the talonid (perpendicular in *Brasilestes* and obtuse in *Deccanolestes*). Also, there is a considerable difference in the size of both teeth: the p3 of *Deccanolestes* is 0.41 mm long, whereas the premolar of *Brasilestes* is 3.5 mm long. It worth mentioning that later revisions of the Cretaceous mammals of India (e.g., ref. [94]) attribute to *Deccanolestes* lower premolars that are morphologically different from the one referred to the type species of the genus [6].

Among the Zalambdalestidae, in *Zalambdalestes lechei* (late Cretaceous of Mongolia) the p1 and p2 are slightly procumbent and the prominent main cusp ends in a posterior cuspule; the unbasined heel of p3 ends in a lingual cuspule; p4 is submolariform [92].

In *Gypsonictops* (late Cretaceous of Canada and the United States), p2 and p3 have no talonid, whereas dp5 and p5 are molariform; although the p4 has a small talonid and is asymmetric in lateral view, there is an anterior cingulum, one to three posterior cuspules, and the posterior wall of the main cusp develops into a crest [47].

The p3 and p4 of *Purgatorius* (early Palaeocene of Canada and the United States) show a small talonid that also ends in a posterior cusp, and a antero-lingual cusp is present in p4 [95].

In *Protungulatum* (late Cretaceous of the United States and early Palaeocene of Canada and the United States), p2 and p3 are more labiolingually compressed and bear an anterior and a posterior cusp flanking the main cusp, while p4 is submolariform [96].

Some similarities were also found between *Brasilestes* and the possible Placentalia that was also collected in the Adamantina Formation [13], especially in the development of the talonid. However, besides the size difference (the length of that p3 is 1.2 mm), the talonid ends in a posterior cusp. Also, the main cusp has an anterior and a posterior crests, is proportionally smaller, and more labiolingually compressed than *Brasilestes*.

Among afrotherians, in the stem *Ocepeia* (middle Palaeocene of Morocco) p3 and p4 are relatively symmetric and develop a trenchant anterior and posterior crest [97]. The premolars are more labiolingually compressed and usually lack talonid in the Afroinsectivora (*sensu* ref. [98]; Afrosoricida + Macroscelidea); the p4 of *Macroscelides* bears a talonid but it is flanked by crests. The teeth in Tubulidentata lack enamel and are compose of dentine tubules; the premolars are ovoid or bilobed, with no cusps (although wear facets may be secondarily present).

Among Eulipotyphla, the lower premolars of *Solenodon* (except for the ultimate, which is molariform) are more symmetric, the main cusp is flanked by anterior and posterior crests and cuspules, and there is no talonid (see ref. [99] for a discussion on premolar homologies). A similar pattern is observed in the premolars of *Talpa*, which are more labiolingually compressed. The single premolar of *Sorex* (p4; [100]) is molariform. The p4 of *Erinaceus* is also molariform, whereas the p2 has no talonid.

Concerning Pholidotamorpha [101], there is no record of teeth in Pholidota. As in *Brasilestes*, the enamel (when present) in Palaeanodonta is reduced to a thin layer [102]. However, the premolars of *Ernanodon* (early Palaeocene of Mongolia and China) are single-rooted and unicuspid, with no talonid [103; 104]. The single preserved premolar (p1) of *Melaniella* (early Palaeocene of Canada) is similar to *Brasilestes* in the asymmetric aspect in lateral view, but the main cusp slopes posteriorly into an unbasined talonid, and is flanked by an anterior and a posterior crest [105]. The p2 and p3 of *Amelotabes* (early Palaeocene of the United States) have a small cusp anterior to the main cusp, and latter tooth also shows a sloping talonid heel [106]. The ultimate premolar of *Tubulodon* (early Eocene of the United States) has a deep basin, one cusp anterior to and two cusps posterior to the main cusp [107]; a thin layer (70–140 µm) of radial enamel with abundant interprimastic matrix covers its entire crown [103]. The p4 of *Mylanodon* (early Palaeocene of the United States) develops a talonid that narrows posterior, as in *Brasilestes*; however, it lacks an anterior crest and the main cusp show a vertical lingual groove [107].

In relation to the Adapisoriculidae, the p4 of *Afrodon* (Palaeocene of Belgium, France, Spain, and Morocco, and early Eocene of Morocco) has a trigonid and one or two talonid cusps [108].

The teeth in the Xenarthra are generally homodont, subcylindric, and the occlusal relief is quickly defined by wearing. Glyptodonts may have more complex pattern, but never develop cusps. Sloths tend to be bilophodont. Enamel is absent, except in the armadillos *Astegotherium* (middle Eocene of Argentina), *Utaetus* (middle Eocene of Argentina), and *Dasypus*, which show a thin layer [54].

**2. Additional data on the enamel**

The SEM images of the enamel (Figure S1-2) show that the thin enamel layer covered most of the crown, except by the tip and the anterior slope of the main cusp. The numerous cracks on the enamel could be due because of postmortem spalling of the tooth exposed to sub-aerial weathering and desiccation [109]. On the other hand, the specimen does not show signs of digestion by predators as a cause of the loss or thinning of the enamel. Considering the fossil fauna of the locality (see above), the most suitable predator of a small to medium-sized mammal like *Brasilestes* would be a crocodile. The crocodile remains are abundant in the Bauru Group and the described forms (~25 species; [110]) range from 0.5 m to 4 m long, approximately. The digestion in modern crocodiles decalcifies tooth tissues, completely removing the enamel (except by residual interdental patches), leaving the organic matrix of the dentine and cement, which decompose within days under aerobic conditions [109]. The presence of a tooth with corroded enamel in a crocodylomorph coprolite from the Bauru Group illustrates such a process [111]; this is not the case of specimen LPRP/USP 0751. Other possible predators in the site would be medium-sized avian and non-avian theropods, but its remains are relatively scarce in the Bauru Group [112; 113]. Digestion by birds (except falconiforms) and mammals does not significantly alter the enamel distribution [109]. Yet, artificial digestion of teeth under experimental conditions shows that digestion corrodes the enamel in a characteristic irregular pattern [114], different than the one observed in *Brasilestes*.


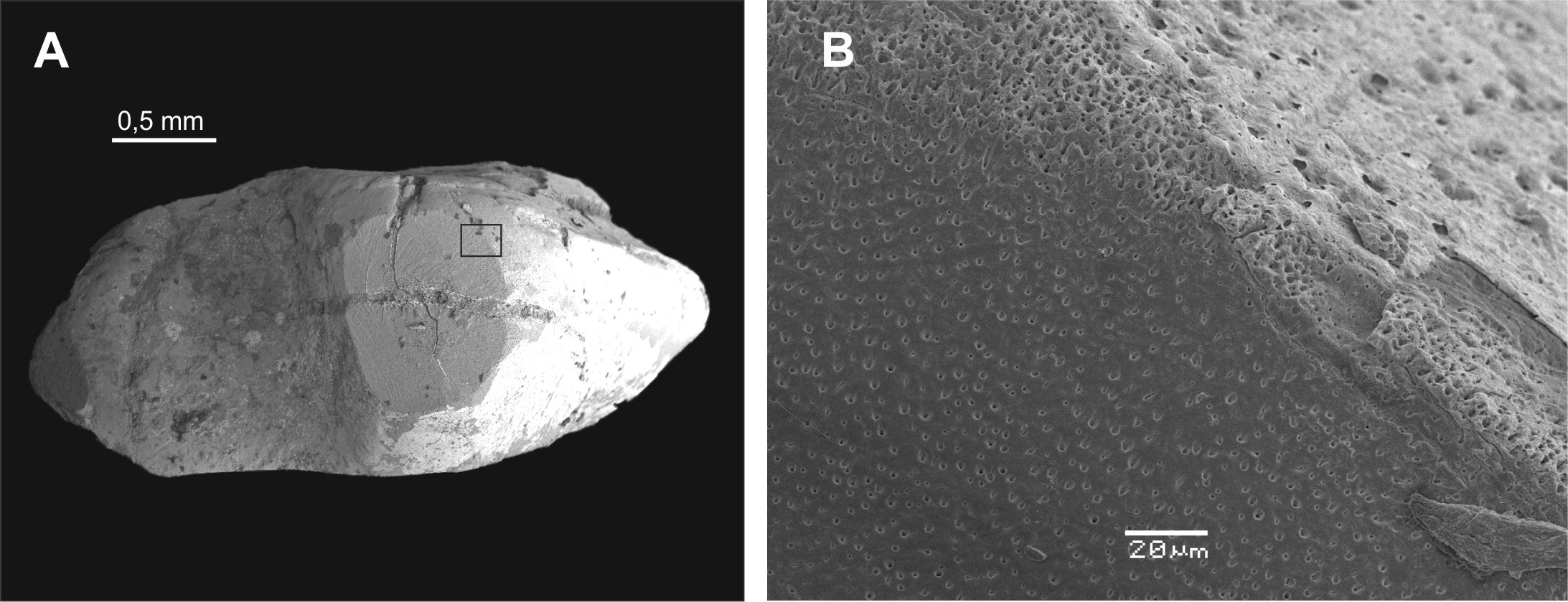


**Figure S1.** SEM images of *Brasilestes stardusti*. A, occlusal view showing the area under study; B, detail of the dentine in the apical zone.

The results of the elemental analyses are similar to previous studies on fossil bones and teeth from several fossil sites [115; 116]. The major elements include oxygen (35.6 wt.%), calcium (29.6 wt.%), and phosphorus (10.2 wt.%). These are components of hydroxyapatite, the main mineral that forms dental enamel and dentin (Figure S3), and may correspond to the original composition of the tooth. The calcium could also originate from paedogenetic precipitation of calcium carbonate during the calcrete formation (see above). This paedogenic event could also be responsible for the great amount of carbon in the sample (19.9 wt.%). Among the minor elements, magnesium (0.5 wt.%), sodium (0.4 wt.%), potassium (0.2 wt.%), and chlorine (0.1 wt.%) may also compose the dentin and enamel [117]. Silicon (1.1 wt.%) and iron (0.5 wt.%), as well as the aluminium (0.97 wt.%) in lesser degree, are more concentrated in the striations on the surface, where the sediment is accumulated (Figure S3), and might have been related to oxidation processes during the current paedogenesis.


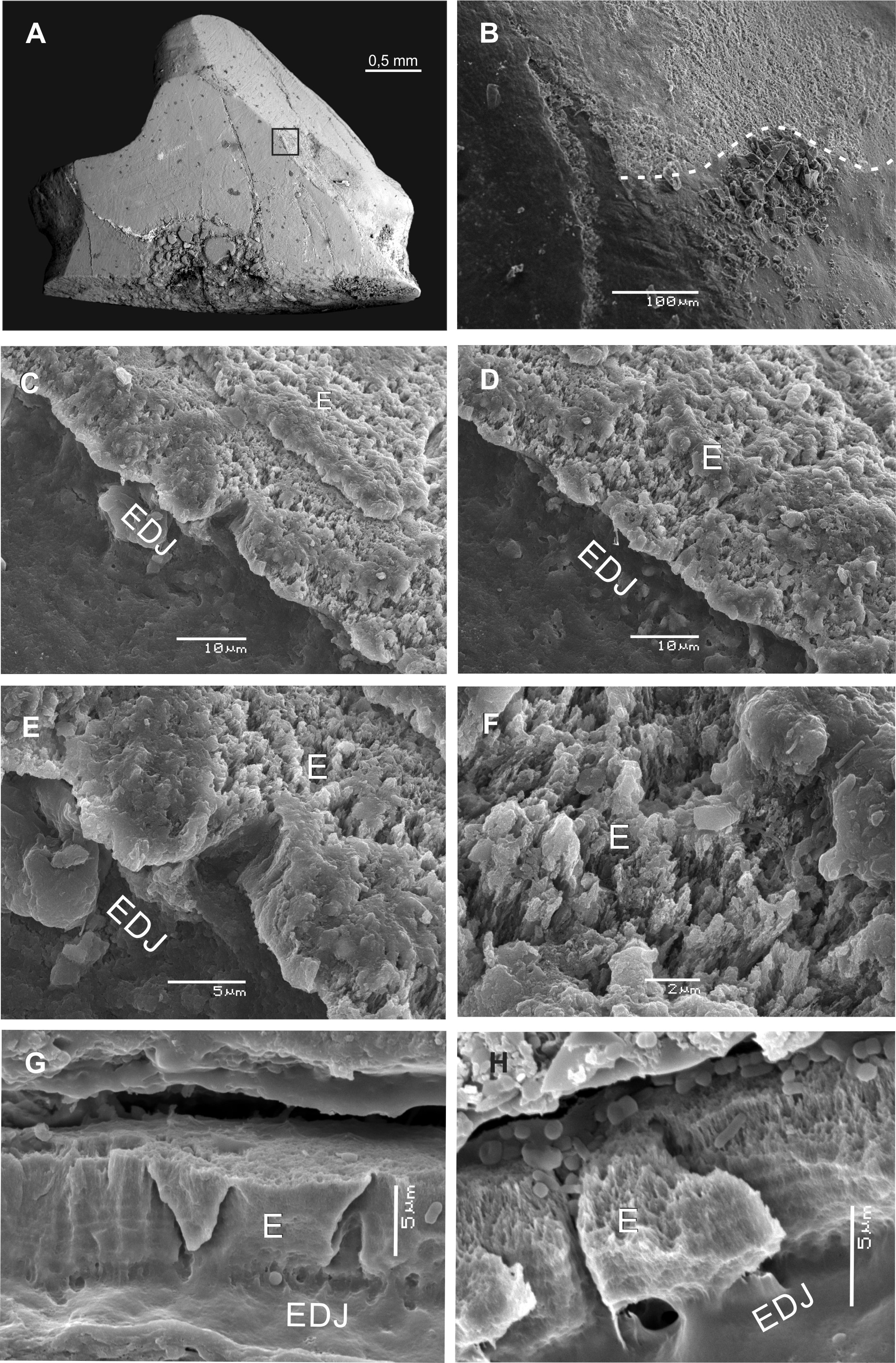


**Figure S2.** SEM images of *Brasilestes stardusti*. A, General labial view; B, Detail of A showing the boundary between enamel and dentine; C-F, thin enamel layer; with no prisms detected; G-H, thin enamel, without prisms, of *Dasypus sabanicola*. E: enamel; EDJ: enamel-dentine junction.


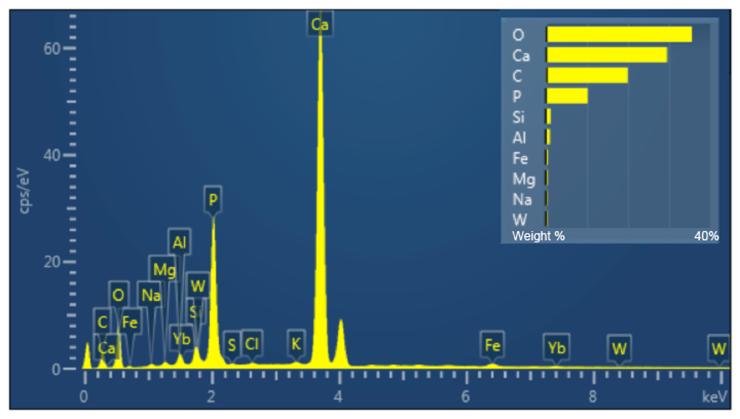


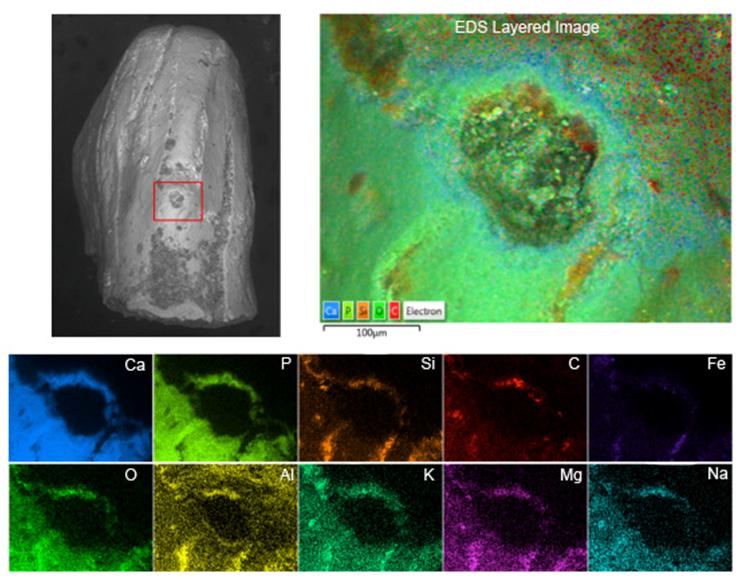


**Figure S3.** EDS elemental analyses spectrum and weight percentage (top) and SEM EDS element distribution maps around the broken anterior cuspule (middle and bottom).

**3. Geological settings**

The Late Cretaceous Bauru Basin occupies an area of about 330,000 km^2^ in south-central Brazil, including parts of São Paulo, Goiás, Mato Grosso do Sul, Mato Grosso, and Minas Gerais states. It was formed by isostatic compensation associated with uplifts in central-eastern South America, which resulted from the activity of the Trindade Mantle Plume [39; 118]. The erosive processes responsible for the current configuration of the basin are related to Tertiary tectonics events: the Serra do Mar and Alto Paranaíba High uplifts at the eastern border, and the faulting related to the genesis of the Pantanal Basin to the west. The basement of the Bauru Basin is mostly comprised of basalts of the Serra Geral Formation, which is part of the Paraná-Etendeka Volcanic Province, dated at ca. 137-127 Ma [119]. The exclusively continental (eolian, lacustrine, fluvial, alluvial) infills of the basin have been traditionally divided into the Caiuá and Bauru groups, but more detailed stratigraphy has been controversial^31,78-79^. Here we follow the scheme of ref. 31, in which the Bauru Group is divided into the Adamantina, Araçatuba, Uberaba, and Marília formations (Figure 1). In alternative proposals, the Adamantina Formation is either integrated into or omitted in lieu of a more complex stratigraphic scheme that variably includes the Vale do Rio do Peixe, Presidente Prudente, and São José do Rio Preto formations [120; 121].

*3.1. Type-locality and -section*

The Buriti Farm, type-locality of *Brasilestes stardusti*, is a well-known fossiliferous site (Figure 1) with abundant *in situ* vertebrate remains recovered since the end of last century [122]. Particularly, it includes a plethora of crocodyliforms such as the baurusuchids *Baurusuchus salgadoensis* [123], *Baurusuchus albertoi* [124], *Gondwanasuchus scabrosus* [125], and *Aplestosuchus sordidus* [126], as well as the sphagesaurid *Caipirasuchus stenognathus* [127]. In addition, snake [128] and theropod dinosaur [129] remains have been identified from the site, along with ichnofossils [123]. These deposits have been mostly ascribed to the Adamantina Formation, following a stratigraphic scheme similar to that employed here [39; 130], in which the Bauru Group is divided into the Adamantina, Araçatuba, Uberaba, and Marília formations. In alternative proposals, the Adamantina Formation is either integrated into or omitted in lieu of a more complex stratigraphic scheme that variably includes the Vale do Rio do Peixe, Presidente Prudente, and São José do Rio Preto formations [121; 122].

As understood by ref. [39], the Adamantina Formation encompasses the totality of the alternating sandstones and mudstones that occur stratigraphically between the underlying Santo Anastácio Formation and the overlying Marília Formation, in western São Paulo [130]. These fine- to medium-grained sandstones are poorly to moderately sorted, reddish, massive or with cross stratifications (foresets up to 3 meters), locally with plane-parallel bedding, ripple marks, mud cracks, and lenticular polymictic conglomerates in upward fining cycles. The type-section (Figure 1) starts with a reddish (10R 5/6), calcareous, sandy palaeosol (P1; Figure S4). Grain size varies from medium to coarse, the rock is poorly sorted and contains subangular quartz clasts. Subordinate conglomeratic facies is composed of angular quartz clasts (0.5-15 mm), clay-rich matrix, and bone fragments. The palaeosol structure is massive, or forming small blocks (up to 4 cm), and roots marks are common. Carbonate occurs locally as cementation or forming nodules. The basal palaeosol yielded the type-material of *Baurusuchus albertoi*, *Aplestosuchus sordidus*, *Gondwanasuchus scabrosus*, and *Caipirasuchus stenognathus*.

**
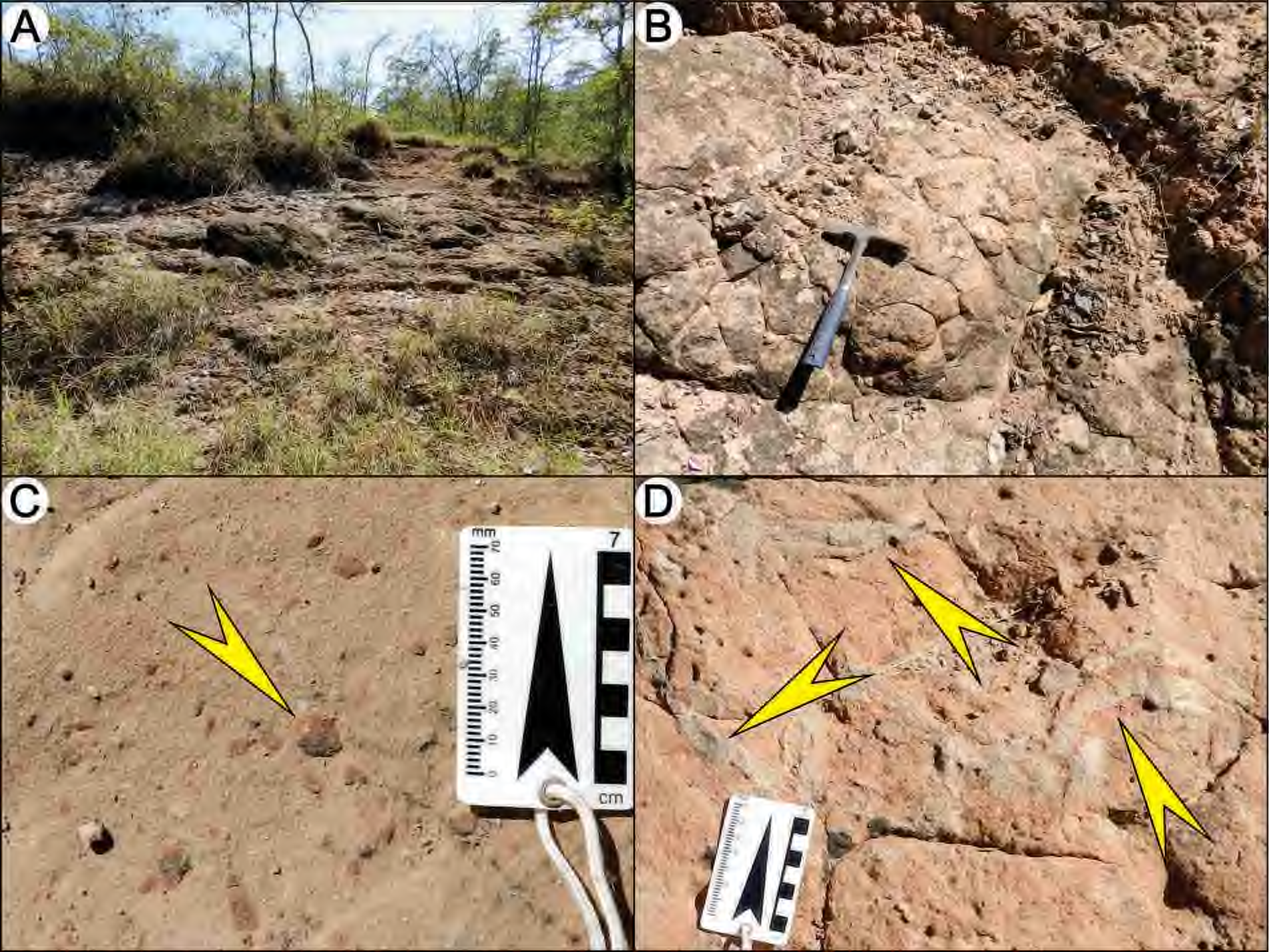
**

**Figure S4.** Palaeosol 1. A, Panoramic view; B, Detail of the block structure; C, Detail of the red clasts of clay; D, Detail of the bioturbation [Photographs by A. Batezelli].

Above the palaeosol there is a succession of fine to medium sandstone layers, up to 12 cm thick each, with a total thickness of 15 meters (TSF; Figure S5). Sandstones extend laterally for hundreds of meters before wedging out. They are upwards fining, with plane-parallel stratifications, and local roots marks (Figure S5B-C). The top of each layer bears ripple marks (10 cm of wave length) and flaser structures (Figure S5D). Two 1.5-meters-thick layers of very fine to fine sandstones (AF1-2; Figure S6) are in the middle and top of the section. These are well-sorted, reddish to yellow sandstones forming low-angle cross-bedding (up to 5 degrees of inclination) with foresets up to 2 meters long.

**
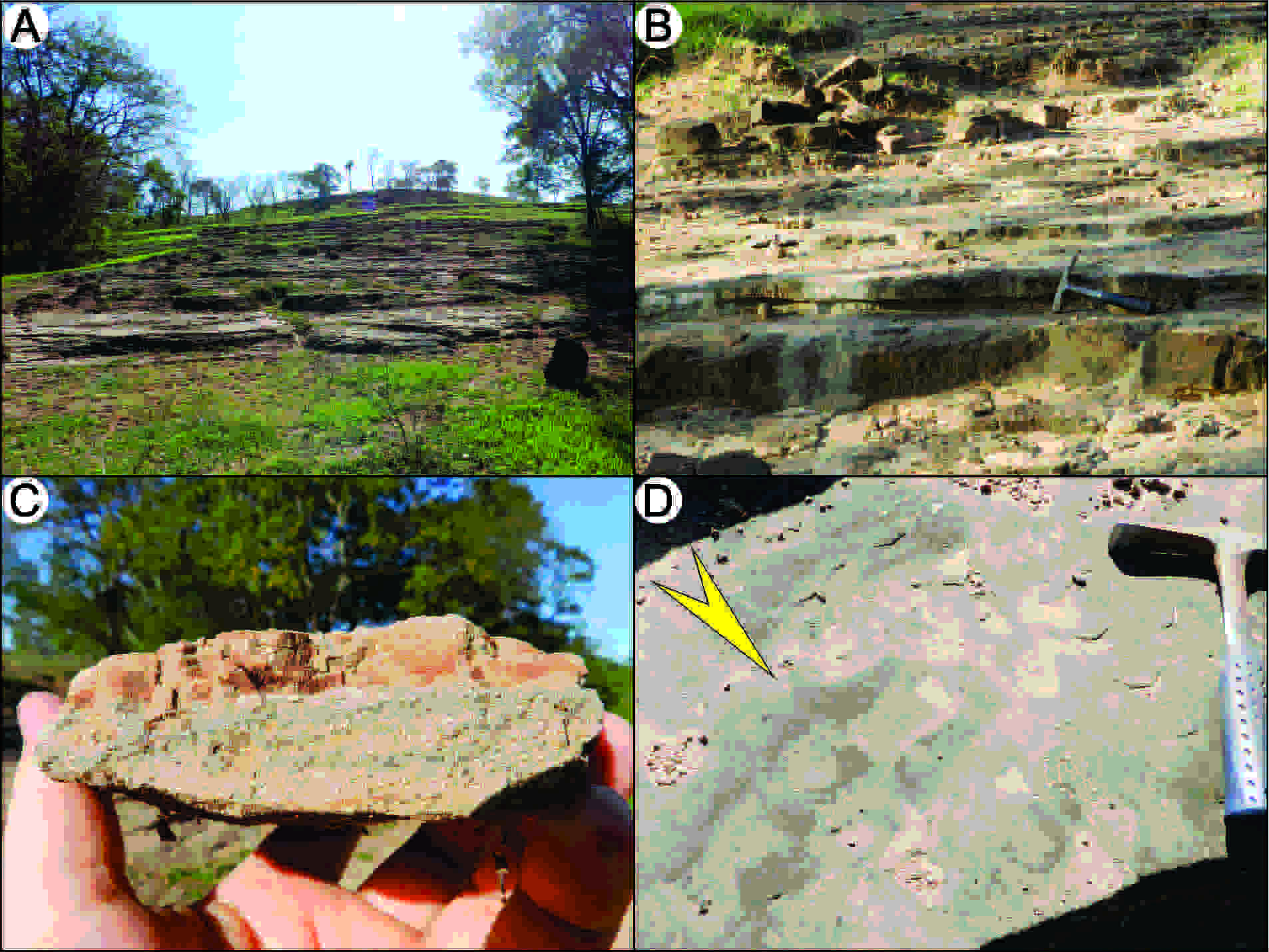
**

**Figure S5.** Tabular Sandstone Facies – TSF. A, Panoramic view; B, Detail of lenticular sandstone; C, Detail of the fine-to-medium sandstones in contact to reddish muddy very fine sandstone in the top; D, Detail of ripples marks in the top of lenticular sandstone (Yellow arrow indicates palaeocurrent from NW to SE) [Photographs by A. Batezelli].

Palaeosol 2 (P2) overlies the Aeolian Facies 1 (AF1) in the upper portion of the section. It is light red (10R 6/6) in colour, has a fine sandy texture, and is poorly sorted, with subangular to angular quartz clasts (3 mm of diameter). This horizon has a calcareous cement and bears macroscopic calcite crystals. It is mostly massive, but subtle plane-parallel stratification is locally present. Block structures and root marks are the most conspicuous features of this upper palaeosol (Figure S6C-D), which yielded the type-material of *Brasilestes stardusti* and *Baurusuchus salgadoensis*, as well as snake remains [129].

**
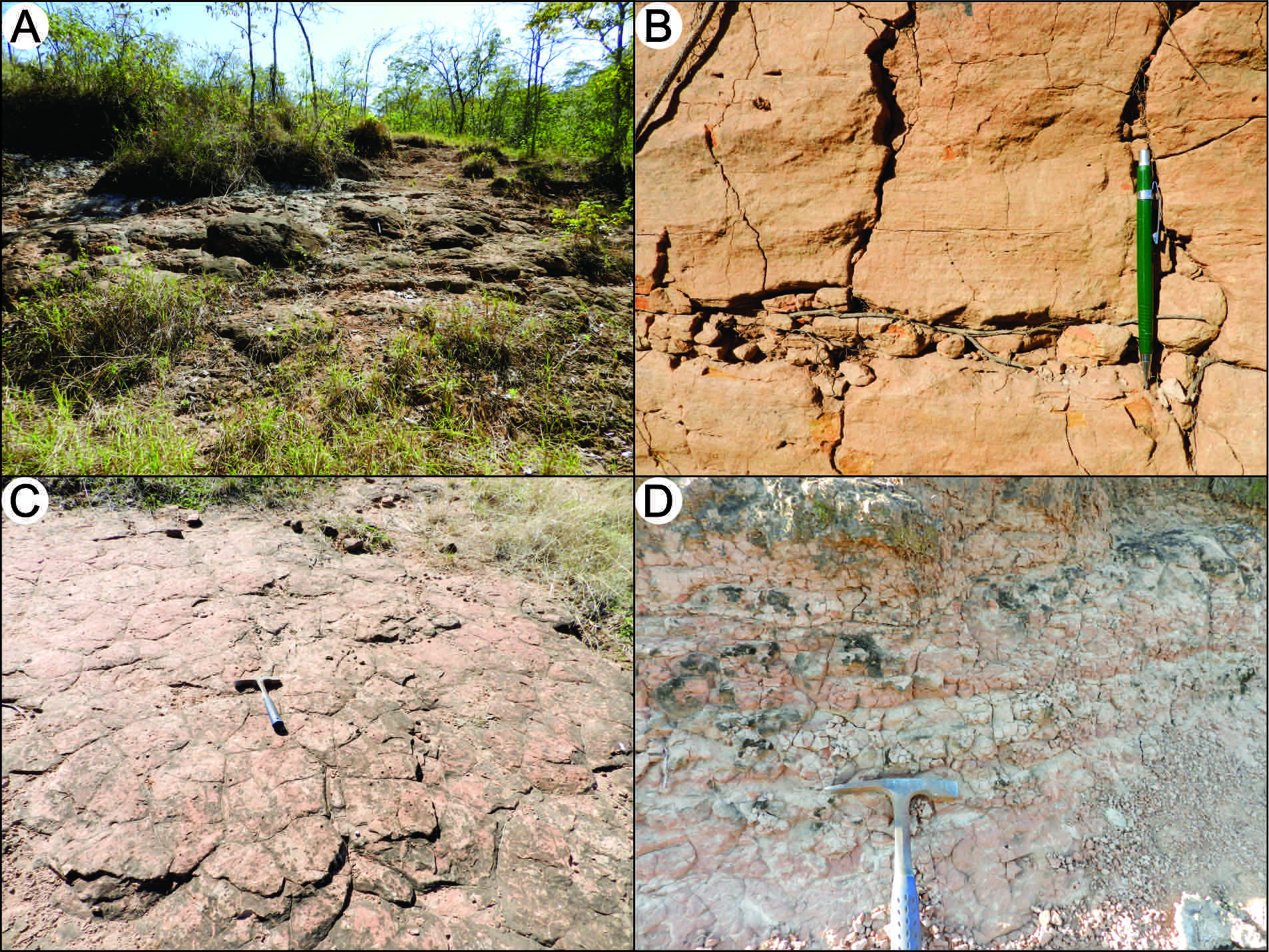
**

**Figure S6.** A, Very fine to fine laminated sandstone (Aeolian Facies 2 - AF2); B, Detail of plane-parallel lamination; C, Detail of the fine-to-coarse Palaeosol 2 (P2) with blocky ped structure; D, Subtle planar-parallel stratification in C horizon of Palaeosol 2 (P2) [Photographs by A. Batezelli].

*3.2. Palaeoenvironmental interpretations*

The depositional facies analysis identified intermittent sedimentation events, probably in fluvial environments under a semi-arid climate. The sandy layers that form the Tabular Sandstone Facies (TSF) indicate subaqueous flows with gradual decrease in energy, which were responsible for the deposition of sheet-like sand bodies – the LS element [131]. Their surface distribution, as well as their tabular geometry and depositional architecture, indicate unconfined fluvial deposits [132]. Palaeocurrent measurements indicate sediment transport from north-northeast to south-southwest, in accordance with previous works (see ref. [133] and references therein), suggesting that the base level was located in the southern portion of the basin.

The depositional model proposed here is similar to the Huesca alluvial system in the Ebro Basin (Spain), where braided river channels are distributary and extend from alluvial fans from the Pyrenees (southern part of the basin) toward the north [134]. The TSF is similar to the thin, wave-rippled, sheet sandstone facies (NW) in distal sectors of fluvial distributary systems (Miocene Luna and Huesca Systems) in northern Spain [135]. Significant north to south facies changes occur within the Bauru Basin [133], including a general decrease in the sediment grain size, changes in the size and architecture of channels, and a higher dispersion of the palaeocurrents. These characteristics define the evolution of an alluvial system dominated by braided rivers (*sensu* ref. [136]) or a distributive fluvial system (*sensu* ref. [137]).

The Aeolian Facies 1 and 2 (AF1 and AF2) occur interlayered with the TSF and indicate the interaction between fluvial and small aeolian dune deposits. Features of Aeolian Facies 1 and 2 suggest wind deposition on a flat surface, above fluvial deposits. The aeolian facies of the Adamantina Formation (i.e. Vale do Rio do Peixe Formation) has been previously recognized (see. ref. [121] and references therein).

Palaeosols have been widely reported from the Bauru Basin, especially in the Marília Formation [133; 138]. Ref. [123] mentioned the occurrence of aridisols interlayered with fluvial deposits, in the fossiliferous intervals of the Adamantina Formation in General Salgado.

The section described in this paper includes two marked palaeosol levels. These bear roots marks, ped structures in small blocks, and have calcareous cement. In some places, subtle plane-parallel stratifications are observed, indicating that the palaeosols developed on the sandy deposits of TSF. These traits allow classifying the palaeosols as pedogenic calcretes [139], which are formed in shallow soil profiles and above the water table, and often have different degrees of development [140].

The occurrence of calcretes indicates semi-arid to arid conditions. Their formation is related to calcium carbonate accumulation in the soils of arid areas, due to the intense evaporation and changes in CO_2_ partial pressure. In modern pedogenic calcretes, calcium carbonate accumulations occur at or close to the surface. Another characteristic that indicates arid to semi-arid conditions is the presence of palygorskite [39; 138]. That mineral is formed under high Mg and Si concentrations, in association to high-pH waters. A maximal rainfall interval between 500 mm and 1000 mm has been suggested [39].

Fossils reported from the studied section are clustered in Palaeosols 1 and 2 (P1 and P2), which were formed on flood deposits. The large number of articulated skeletons, as well as their good preservation, indicates that the organisms did not suffer much transport, as described from the Adamantina Formation in Minas Gerais [138]. The palaeosols indicate that two non-deposition episodes intervened the fluvial sedimentation (TSF), when the deposits were exposed to weathering and paedogenesis. In this time interval, calcification process generated the calcretes in which the skeletons were preserved. The evolution of deposition and paedogenesis in the section described in General Salgado-SP is shown in the model of Figure S7.


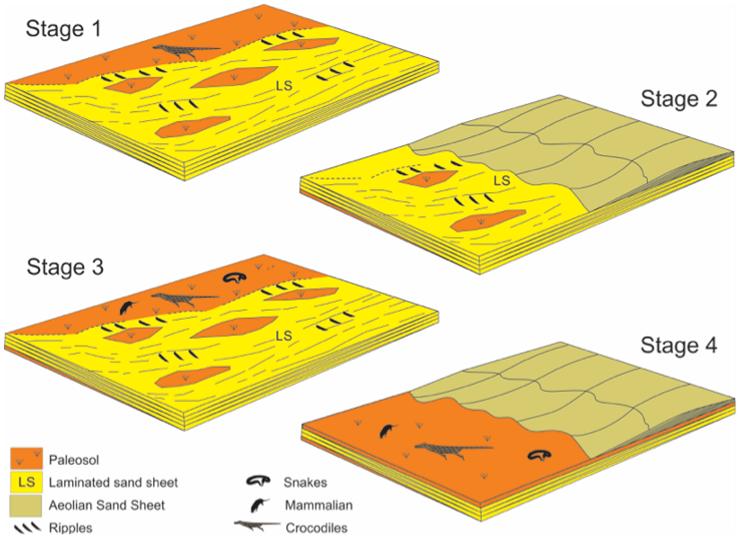


**Figure S7.** Evolutionary model for the deposits of the Adamantina Formation in the study.

**4. U-Pb geochronology**

The LA-ICP-MS ^206^Pb/^238^U dates have an average 2σ uncertainty of 2.6% and range from ca. 3.3 Ga to 85.2 ± 2.7 Ma, indicating a highly mixed detrital zircon population. The ID-TIMS ^206^Pb/^238^U dates are characterized by 2σ uncertainties on the order of 0.2% and range from 620.4 ±1.6 Ma to 87.782 ± 0.062 Ma. The youngest two high-precision ID-TIMS analyses (z17 and z18*) overlap within uncertainty (and with the youngest five LA-ICP-MS dates), probably representing the youngest zircons that exist in the sample (Figure S8). The data are not sufficient to calculate a weighted mean date, therefore, the youngest ID-TIMS date of 87.782 ± 0.062 Ma (± 0.12 Ma including total propagated uncertainties) best represents the maximum age of deposition of the fossil-bearing bed.


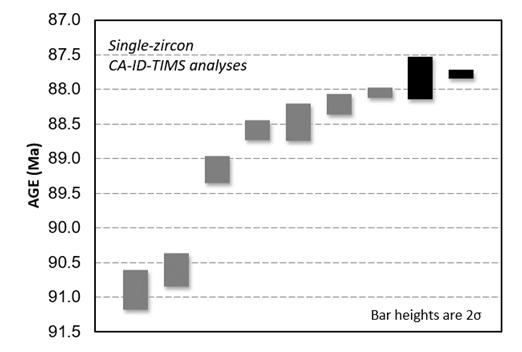


**Figure S8.** Date distribution plot of the youngest high-precision ID-TIMS U-Pb zircon analyses from the *Brasilestes stardusti* bed. Each bar represents a single zircon analysis. Solid bars represent the analyses used to derive the maximum depositional age of the sample.

**Table S1.** U-Pb LA-ICP-MS data for the analysed zircons from the *Brasilestes stardusti* bed.

*Youngest analyses that were also analysed by the ID-TIMS method.

**Table S2.** U-Pb ID-TIMS data for the analysed zircons from the *Brasilestes stardusti* bed.

(a) Thermally annealed and pre-treated single zircon. Data used in age calculation are shown in bold. Asterix signifies zircons analyzed also by the LA-ICP-MS method.

(b) Total common-Pb in analyses.

(c) Measured ratio corrected for tracer and fractionation only.

(d) Radiogenic Pb.

(e) Corrected for fractionation, tracer and blank. Also corrected for initial Th/U disequilibrium using radiogenic 208Pb and Th/U[magma] = 2.8

Mass-dependent fractionation correction of 0.25%/amu ± 0.04%/amu (atomic mass unit) was applied to single-collector Daly analyses on Sector 54 and 0.18%/amu ± 0.04%/amu on X62 instruments.

All common Pb assumed to be laboratory blank. Total procedural blank less than 0.1 pg for U.

Blank isotopic composition: 206Pb/204Pb = 18.42 ± 0.35, 207Pb/204Pb =15.36 ± 0.23, 208Pb/204Pb = 37.46 ± 0.74.

Corr. coef. = correlation coefficient.

Dates calculated using the decay constants of Jaffey et al. (1971).

**5. Supplementary Information References**

1. Wible JR, Rougier GW, Novacek MJ, Asher RJ. 2009. The eutherian mammal *Maelestes gobiensis* from the Late Cretaceous of Mongolia and the phylogeny of Cretaceous Eutheria. *Bull Am Mus Nat Hist* **327**, 1–123.
2. Flynn JJ, Parrish JM, Rakotosamimanana B, Simpson WF, Wyss AR. 1999. A middle Jurassic mammal from Madagascar. *Nature* **401**, 57–60.
3. Rich, T. H. *et al.* 1997. A tribosphenic mammal from the Mesozoic of Australia. *Science* **278**, 1438–1442.
4. Rich, T. H. *et al.* 2001. A second tribosphenic mammal from the Mesozoic of Australia. *Rec. Queen Vic. Mus.* **110**, 1–9.
5. Rauhut OW, Martin T, Ortiz-Jaureguizar E, Puerta P. 2002. A Jurassic mammal from South America. *Nature* **416**, 165–168.
6. Kielan-Jaworowska Z, Cifelli RL, Luo ZX. 2002. Dentition and relationships of the Jurassic mammal *Shuotherium*. *Acta Palaeontol Pol* **47**, 479–486.
7. Krause DW *et al.* 2014. First cranial remains of a gondwanatherian mammal reveal remarkable mosaicism. *Nature* **515**, 512–517.
8. Martin T. 1997. Tooth replacement in Late Jurassic Dryolestidae (Eupantotheria, Mammalia). *J Mammal Evol* **4**, 1–18.
9. Gelfo JN, Pascual R. 2001. *Peligrotherium tropicalis* (Mammalia, Dryolestida) from the early Paleocene of Patagonia, a survival from a Mesozoic Gondwanan radiation. *Geodiversitas* **23**, 369–379.
10. Rougier GW, Apesteguía, S., & Gaetano, L. C. Highly specialized mammalian skulls from the Late Cretaceous of South America. *Nature* **479**, 98–102 (2011).
11. Martin T. 2002. New stem-lineage representatives of Zatheria (Mammalia) from the Late Jurassic of Portugal. *J Vert Paleontol* **22**, 332–348.
12. Davis BM. 2011. A novel interpretation of the tribosphenidan mammal *Slaughteria* *eruptens* from the Lower Cretaceous Trinity Group, and implications for dental formula in early mammals. *J Vert Paleontol* **31**, 676–683.
13. Kielan-Jaworowska Z, Dashzeveg D. 1989. Eutherian mammals from the Early Cretaceous of Mongolia. *Zool Scr* **18**, 347–355.
14. Sigogneau-Russell D., Dashzeveg, D. & Russell, D. E. Further data on *Prokennalestes* (Mammalia, Eutheria *inc. sed.*) from the Early Cretaceous of Mongolia. *Zool Scr* **21**, 205–209 (1992).
15. Cifelli RL. 1999. Tribosphenic mammal from the North American early Cretaceous. *Nature* **401**, 363–366.
16. Archibald JD, Averianov A. 2012. Phylogenetic analysis, taxonomic revision, and dental ontogeny of the Cretaceous Zhelestidae (Mammalia: Eutheria). *Zool J Linn Soc* **164**, 361–426.
17. Fox RC. 1979. Mammals from the Upper Cretaceous Oldman Formation, Alberta. III. Eutheria. *Can J Earth Sci* **16**, 114–125.
18. Averianov AO, Archibald JD. 2013. New material and reinterpretation of the Late Cretaceous eutherian mammal *Paranyctoides* from Uzbekistan. *Acta Palaeontol Pol* **58**, 17–23.
19. Kielan-Jaworowska Z. 1969. Preliminary data on the Upper Cretaceous eutherian mammals from Bayn Dzak, Gobi Desert. *Palaeontol Pol* **19**, 171–191.
20. Prasad GVR, Khajuria CK. 1990. A record of microvertebrate fauna from the intertrappean beds of Naskal, Andhra Pradesh. *J Palaeontol Soc India* **35**, 151–161.
21. Verma O. 2008. *Origin, diversity, phylogenetic and palaeobiogeographic relationships of Cretaceous mammals of India* (Unpublished Ph.D. Thesis, University of Jammu, India).
22. Clemens WA. 1974. *Purgatorius*, an early paromomyid primate (Mammalia). *Science* **184**, 903–905.
23. Sloan RE, Van Valen L. 1965. Cretaceous mammals from Montana. *Science* **148**, 220–227.
24. Gheerbrant E, Amaghzaz M, Bouya B, Goussard F, Letenneur C. 2014. *Ocepeia* (Middle Paleocene of Morocco): the oldest skull of an afrotherian mammal. *PloS One* **9**, e89739.
25. Seiffert ER. 2007. A new estimate of afrotherian phylogeny based on simultaneous analysis of genomic, morphological, and fossil evidence. *BMC Evol Biol* **7**, 224.
26. Wible JR. 2008. On the cranial osteology of the Hispaniolan solenodon, *Solenodon paradoxus* Brandt, 1833 (Mammalia, Lipotyphla, Solenodontidae). *Ann Carnegie Mus* **77**, 321–402.
27. Hutterer R. 2005. Homology of unicuspids and tooth nomenclature in shrews. *Advances in the Biology of Shrews*, 397–404.
28. Gaudin TJ, Emry RJ, Wible JR. 2009. The phylogeny of living and extinct pangolins (Mammalia, Pholidota) and associated taxa: a morphology based analysis. *J Mammal Evol* **16**, 235–305.
29. Kalthoff DC, Rose KD, von Koenigswald W. 2011. Dental microstructure in *Palaeanodon* and *Tubulodon* (Palaeanodonta) and bioerosional tunneling as a widespread phenomenon in fossil mammal teeth. *J Vert Paleontol* **31**, 1303–1313.
30. Radinsky L, Ting SY. 1984. The skull of *Ernanodon*, an unusual fossil mammal. *J Mammal* **65**, 155–158.
31. Kondrashow P, Agadjanian AK. 2012. A nearly complete skeleton of *Ernanodon* (Mammalia, Palaeanodonta) from Mongolia: morphofunctional analysis. *J Vert Paleontol* **32**, 983–1001.
32. Fox RC. 1984. *Melaniella timosa* n. gen. and sp., an unusual mammal from the Paleocene of Alberta, Canada. *Can J Earth Sci* **21**, 1335–1338.
33. Rose KD. 1978. A new Paleocene epoicotheriid (Mammalia), with comments on the Palaeanodonta. *J Paleontol* **52**, 658–674.
34. Secord R, Gingerich PD, Bloch JI. 2002. *Mylanodon rosei*, a new Metacheiromyid (Mammalia: Palaeanodonta) from the Late Tiffanian (Late Paleocene) of Northwestern Wyoming. *Contrib Mus Paleontol Univ Michigan* **30**, 385–399.
35. Gheerbrant E, Russell DE. 1989. Presence of the genus *Afrodon* [Mammalia, Lipotyphla (?), Adapisoriculidae] in Europe; new data for the problem of trans-Tethyan relations between Africa and Europe around the K/T boundary. *Palaeogeog Palaeoclimatol Palaeoecol* **76**, 1–15.
36. Fisher JA, Nichols GJ, Waltham DA. 2007. Unconfined flow deposits in distal sectors of fluvial distributary systems: examples from the Miocene Luna and Huesca Systems, northern Spain. *Sediment Geol* **195**, 55–73.
37. Martinelli AG, Teixeira VPA. 2015. The Late Cretaceous vertebrate record from the Bauru Group in the Triângulo Mineiro, southeastern Brazil. *Bol Geol Min* **126**, 129–158.
38. Souto PDF. 2010. Crocodylomorph coprolites from the Bauru basin, upper Cretaceous, Brazil. *Bull New Mexico Mus Nat Hist Sci***51**, 201–208.
39. Alvarenga H, Nava WR. 2005. Aves Enantiornithes do Cretáceo Superior da Formação Adamantina do Estado de São Paulo, Brasil. *II Congreso* *Latinoamericano de Paleontología de Vertebrados, Rio de Janeiro* **20**.
40. Marsola JCA, Grellet-Tinner G, Montefeltro FC, Sayão JM, Hsiou AS, Langer MC. 2014. The first fossil avian egg from Brazil, *Alcheringa* **38**, 563–567.
41. Fernández‐Jalvo Y, Andrews P, Sevilla P, Requejo V. 2014. Digestion versus abrasion features in rodent bones. *Lethaia* **47**, 323–336.
42. Previtera E, D'angelo JA, Mancuso AC. 2013. Preliminary chemometric study of bone diagenesis in Early Triassic cynodonts from Mendoza, Argentina. *Ameghiniana* **50**, 460–468.
43. Smith T, Codrea V. 2015. Red iron-pigmented tooth enamel in a multituberculate mammal from the Late Cretaceous Transylvanian “Haţeg Island”. *PloS One* **10**, e0132550.
44. Dauphin Y, Williams CT. 2007. The chemical compositions of dentine and enamel from recent reptile and mammal teeth—variability in the diagenetic changes of fossil teeth. *CrystEngComm* **9**, 1252–1261.
45. Gibson SA, Thompson RN, Leonardos OH, Dickin AP, Mitchell JG. 1995. The late cretaceous impact of the trindade mantle plume: evidence from large volume, Mafic, Potassic Magmatism in SE Brazil. *J Petrol* **36**, 189–229.
46. Turner S, Regelons M, Kelley S, Jawkesworth C, Mantovani MSM. 1994. Magmatism and continental break-up in the South Atlantic: high precision geochronology. *Earth Planet Sci Lett* **121**, 333–348.
47. Fernandes LA, Ribeiro CMM. 2015. Evolution and palaeoenvironment of the Bauru Basin (upper Cretaceous, Brazil). *J S Am Earth Sci* **61**, 71–90.
48. Menegazzo MC, Catuneanu O, Chang HK. 2016. The South American retroarc foreland system: The development of the Bauru Basin in the back-bulge province. *Mar Petrol Geol* **73**, 131–156.
49. Carvalho IS *et al.* 2010. Répteis Fósseis de General Salgado, SP-Registro de transformações ambientais na Bacia Bauru durante o Cretáceo. *SIGEP* **53**.
50. Carvalho IS, Campos ACA, Nobre PH. 2005. *Baurusuchus salgadoensis*, a new Crocodylomorpha from the Bauru Basin (Cretaceous), Brazil. *Gondwana Res* **8**, 11–30.
51. Nascimento PM, Zaher H. 2010. A new species of *Baurusuchus* (Crocodyliformes, Mesoeucrocodylia) from the Upper Cretaceous of Brazil, with the first complete postcranial skeleton described for the family Baurusuchidae. *Pap Avulsos Zool* **50**, 323–361.
52. Marinho TS, Iori FV, Carvalho IS, Vasconcellos FM. 2013. *Gondwanasuchus scabrosus* gen. et sp. nov., a new terrestrial predatory crocodyliform (Mesoeucrocodylia: Baurusuchidae) from the Late Cretaceous Bauru Basin of Brazil. *Cretaceous Res* **44**, 104–111.
53. Godoy PL, Montefeltro FC, Norell MA, Langer MC. 2014. An additional baurusuchid from the Cretaceous of Brazil with evidence of interspecific predation among Crocodyliformes. *PLoS One*, **9** e97138.
54. Pol D. *et al.* 2014. A new notosuchian from the Late Cretaceous of Brazil and the phylogeny of advanced notosuchians. *PLoS One* **9**, e93105.
55. Zaher HD, Langer MC, Fara E, Carvalho IS, Arruda JT. 2003. A mais antiga serpente (Anilioidea) brasileira: Cretáceo Superior do Grupo Bauru, General Salgado, SP. *Paleontol Destaque* **44**, 50–51.
56. Vasconcellos FM, Marinho TS, Iori FV, Morato L. 2008. First dinosaur remains from General Salgado surroundings (Adamantina Formation, Bauru Basin - Late Cretaceous), São Paulo state, Brazil. *Paleonotícias Boletim Especial* 47–48.
57. Soares PC, Landim PMB, Fulfaro VJ, Sobreiro Neto AF. 1980. Ensaio de caracterização estratigráfica do Cretáceo no Estado de São Paulo: Grupo Bauru. *Rev Bras Geoc* **10**, 177–185.
58. Miall AD. 2014 Fluvial depositional system. Springer-Verlag, Berlin.
59. North CP, Davidson SK. 2012. Unconfined alluvial flow processes: recognition and interpretation of their deposits, and the significance for palaeogeographic reconstruction. *Earth-Sci Rev* **111**, 199–223.
60. Batezelli A, Ladeira FSB. 2016. Stratigraphic framework and evolution of the Cretaceous continental sequences of the Bauru, Sanfranciscana, and Parecis basins, Brazil. *J South Am Earth Sci* **65**, 1–24.
61. Hirst JPP. 1991. Variations in alluvial architecture across the Oligo-Mioceno Huesca Fluvial System Ebro Basin, Spain. *SEPM* **3**, 111–121.
62. Fisher JA, Nichols GJ, Waltham DA. 2007. Unconfined flow deposits in distal sectors of fluvial distributary systems: examples from the Miocene Luna and Huesca Systems, northern Spain. *Sediment Geol* **195**, 75–90.
63. Stanistreet IG, McCarthy TS. 1993. The Okavango Fan and the classification of sub aerial fan systems. *Sediment Geol* **85**, 115–133.
64. Hartley AJ *et al.* 2012. Soil development on modern distributive fluvial systems: preliminary observations with implications for interpretation of paleosols in the rock record. *SEPM Special Publ* **104**, 149–158.
65. Marsola JCA, Batezelli A, Montefeltro FC, Grellet-Tinner G, Langer MC. 2016. Palaeoenvironmental characterization of a crocodilian nesting site from the Late Cretaceous of Brazil and the evolution of crocodyliform nesting strategies. *Palaeogeog Palaeoclimatol Palaeoecol* **457**, 221–232.
66. Wright P, Tucker ME. 1991. Calcretes. Blackwell Scientific Publications, Oxford.
67. Alonso-Zarza AM. 2003. Paleoenveironmental significance of palustrine carbonates and calcretes in the geological record. *Earth Sci Rev* **60**, 261–298.
